# Supplementary material for: The genome sequence of an Entiminae weevil, Polydrusus pterygomalis Boheman, 1840
Source: Wellcome Open Res. 2024 Sep 18;9:528. [Version 1] doi: 10.12688/wellcomeopenres.23048.1 (PMC11494282; doi:10.12688/wellcomeopenres.23048.1)
Supplement: Peer review history

# Reviewer report: Reviewer response for version 1

**DOI:** 10.21956/wellcomeopenres.25382.r103296

**Published:** 2024-10-21

**Authors:** Manee M Manee

**Affiliations:** 1 King Abdulaziz City for Science and Technology, Riyadh, Saudi Arabia

A high-quality genome assembly of the Entiminae weevil *Polydrusus pterygomalis *has been produced as part of the Darwin Tree of Life Project. The assembly covers 1,051.50 mega bases, with most sequences scaffolded into 11 chromosomal pseudomolecules, including the X chromosome, and the mitochondrial genome was fully assembled. The genome has a BUSCO completeness score of 99.1%, indicating its high quality and reliability. This genome contributes to understanding the genetic makeup of *P. pterygomalis*, aiding research in genetics, taxonomy, and biodiversity conservation, with potential impacts on pest management and ecological studies. I am happy with the current version of the article.

Are sufficient details of methods and materials provided to allow replication by others?

Yes

Is the rationale for creating the dataset(s) clearly described?

Yes

Are the datasets clearly presented in a useable and accessible format?

Yes

Are the protocols appropriate and is the work technically sound?

Yes

Reviewer Expertise:

bioinformatics, genomics, and evolutionary biology

I confirm that I have read this submission and believe that I have an appropriate level of expertise to confirm that it is of an acceptable scientific standard.

# Reviewer report: Reviewer response for version 1

**DOI:** 10.21956/wellcomeopenres.25382.r100250

**Published:** 2024-10-12

**Authors:** Elina Immonen, Milena Trabert

**Affiliations:** 1 Ecology and Genetics, Uppsala University, Uppsala, Sweden; 2 Uppsala University Department of Ecology and Genetics (Ringgold ID: 193212), Uppsala, Uppsala, Sweden

The Data Note presents a genome assembly of a weevil *Polydrusus pterygomalis, *released for reuse as part of the Darwin Tree of Life project. The genome covers around 1Gb and is organised into 11 chromosomal-level scaffolds, including the X chromosome identified by sequence homology. Female specimen was sequenced and the Y chromosome is thus missing from the assembly. The assembly is of high quality, exceeding all the benchmark criteria. The mitochondrial genome with a length of about 21kb is assembled. The methods are clearly explained. We recommend indexing this article.

Are sufficient details of methods and materials provided to allow replication by others?

Yes

Is the rationale for creating the dataset(s) clearly described?

Yes

Are the datasets clearly presented in a useable and accessible format?

Yes

Are the protocols appropriate and is the work technically sound?

Yes

Reviewer Expertise:

Evolutionary genetics and genomics

We confirm that we have read this submission and believe that we have an appropriate level of expertise to confirm that it is of an acceptable scientific standard.
